# Supplementary material for: A Global Screen for Assembly State Changes of the Mitotic Proteome by SEC-SWATH-MS
Source: Cell Syst. 2020 Feb 26;10(2):133–155.e6. doi: 10.1016/j.cels.2020.01.001 (PMC7042714; doi:10.1016/j.cels.2020.01.001)

Q9Y248 | PSF2_HUMAN | GINS2 PSF2 CGI−122 DC5 HSPC037
Monomer MW [kDa]: 21.428 Monomer expected elution fraction: 54

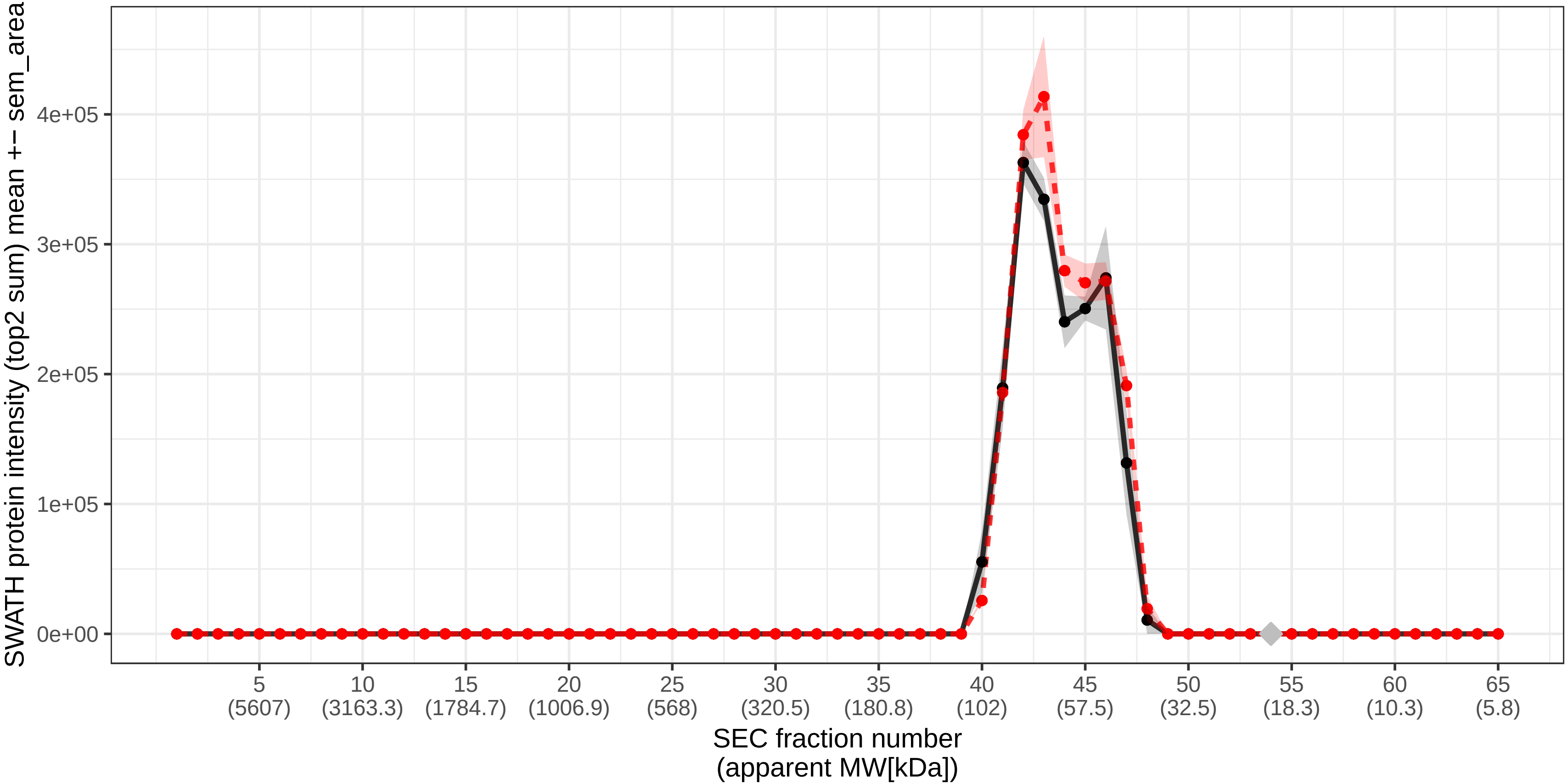

Supplement: Data S1. SEC-SWATH-MS Protein Chromatograms, Related to Figure 1 [file mmc6.zip › SECchrom_Q9Y248_PSF2_HUMAN_GINS2_PSF2_CGI-122_DC5_HSPC037.pdf]
